# Pretreatment with a CRF antagonist amplifies feeding inhibition induced by fourth ventricular cocaine- and amphetamine-regulated transcript peptide

**DOI:** 10.1186/s12868-019-0494-8

**Published:** 2019-03-18

**Authors:** Ulrika Smedh, Karen A. Scott, Timothy H. Moran

**Affiliations:** 1 The Surgical Metabolic Research Laboratory, Department of Surgery, Institute of Clinical Sciences, Sahlgrenska Academy University of Gothenburg, and Sahlgrenska University Hospital, 413 45 Gothenburg, Sweden; 2 Department of Psychiatry and Behavioral Science, The Johns Hopkins University School of Medicine, Baltimore, USA

**Keywords:** Rat, Dorsal hindbrain, Food intake

## Abstract

**Background:**

Pre-treatment with the corticotropin-releasing factor antagonist α-helical CRF9-41 prevents inhibition of gastric emptying by cocaine-and amphetamine-regulated transcript peptide at a dorsal hindbrain level, but its inhibition of sucrose intake is not affected. This is suggestive of separable underlying mechanisms of action in the caudal brainstem for cocaine-and amphetamine-regulated transcript peptide with regard to food intake and gastrointestinal functions. Here we further examine cocaine-and amphetamine-regulated transcript peptide—corticotropin-releasing factor receptor interactions in caudal brainstem controls of solid food intake. Injections of combinations of vehicle, cocaine-and amphetamine-regulated transcript peptide (0.5 μg or 1 μg) or α-helical CRF9-41 were given into the fourth cerebral ventricle of rats. Nocturnal solid food intake was recorded over 22 h.

**Results:**

Pre-treatment with α-helical CRF9-41 into the fourth ventricle significantly increased the responsivity to cocaine-and amphetamine-regulated transcript peptide on hypophagia. In a separate control experiment, α-helical CRF9-41 pre-treatment blocked CRF-induced food intake inhibition indicative of its antagonistic effectiveness.

**Conclusions:**

We conclude that an endogenous Corticotropin-releasing factor agonist may modulate suppression of food intake caused by cocaine-and amphetamine-regulated transcript peptide at a dorsal hindbrain level in the absence of stress. A potential caudal brainstem mechanism whereby cocaine-and amphetamine-regulated transcript peptide effects on food intake is attenuated via corticotropin-releasing factor receptor activity causing tonic inhibition, is suggested.

## Background

We tested the hypothesis that cocaine- and amphetamine-regulated transcript-derived peptides (CARTp) can act at a caudal brainstem level to modify food intake via a CRF-receptor modulated mechanism. CARTp are endogenous ligands that act in the brain and contribute to food intake regulation [1–3] and gastro-motor controls [4, 5]. Although functional studies have identified the dorsal hindbrain as a region of importance for CARTp effects on food intake and gastrointestinal function, the putative receptors and receptor sites for CARTp are not yet known. Evidence suggest that the brainstem sites and controlling mechanisms for CARTp to affect food intake versus gastro-motor controls may be separate, however [2, 3, 5–7].

CARTp appears to interact with a variety of neuropeptides, among these Corticotropin-releasing factor (CRF) [4, 5, 8, 9]. The dorsal hindbrain has a high density of CART-containing cells and nerves in multiple areas which are associated with controls for food intake and body weight, including the nuclei of the solitary tract, dorsal Raphe, Edinger–Westphal, as well as the locus coeruleus and the area postrema [10, 11], all of which also express CRF receptors, express endogenous CRF receptor agonists or are within the terminating field of CRF-containing fibers. Together, this provides anatomical support for possible dorsal hindbrain interactions of CRF and CARTp. Functional studies show that CRF and CARTp indeed display many similar effects at a caudal brainstem level and support the possibility of an interaction, at least with regard to gastrointestinal functions [4, 5, 7, 12]. For example, similar to CARTp [2, 3, 7, 13], fourth i.c.v. injections of CRF or urocortin causes hypophagia [14, 15]. Based on the observations that fourth i.c.v. or intracisternal CARTp-induced inhibition of gastromotor function and acid secretion, respectively, are blocked by the CRF antagonist alpha-helical CRF 9-41 (αCRF) we previously investigated whether the hypophagic effect of CARTp elicited at a dorsal hindbrain level could be similarly mediated. We found however, that not only did αCRF fail to antagonize the effect of CARTp-induced suppression of sucrose intake, but rather showed some tendency towards a synergistic inhibitory effect [7]. Since CARTp inhibits drinking as well [3], it is possible that a sucrose or milk drinking test may be a less sensitive model to detect a full range of CARTp–CRF interactions on food intake. For the present investigations, we therefore used solid food intake as paradigm to test the hypothesis that caudal brainstem CARTp effects on solid food ingestion is modulated via a CRF-receptor mechanism. Given that putative CARTp receptor sites are not yet known, and multiple caudal brainstem substrates could be involved as site(s) of action, we used the fourth i.c.v. route of administration and pre-treated rats with the un-selective CRF receptor antagonist alpha-helical-CRF9-41, prior to administration of CARTp at doses previously shown subthreshold, or effective, for a hypophagic effect in the rat.

## Materials and methods

### Animals

Male Sprague–Dawley rats (Charles River Laboratories) which were 10–12 weeks of age and weighing between 400 and 450 g were used. The animals were housed singly in hanging cages with wire mesh floors, and had free access to chow (Prolab RMH 1000) and tap water under conditions of controlled temperature (20 ± 1 °C) and humidity, on a 12:12 h light cycle (lights off 1 p.m.–1 a.m.). According to protocol, the animals were euthanized with a lethal dose of Euthasol^®^, i.p. at the end of the study. In addition, the abdominal aorta was cut after death had occurred. Rats were weighed and checked for health daily. All animal experimental protocols were approved by the Institutional Animal Care and Use Committee at Johns Hopkins University, Baltimore.

### Surgery

Prior to surgery, the rats were anesthetized with a 3:4 mixture of Xylazine (20 mg/ml) and Ketamine (100 mg/ml), injected i.m. The surgeries were performed under aseptic conditions. The animals were placed in a stereotaxic frame and the scull was exposed. A chronic guide cannula (10.0 mm × 27 G) aimed at the fourth ventricle was implanted 2.6 mm anterior to the occipital crest in the midsagittal line after trepanation, as previously described [16]. These coordinates had been determined for rats of the present size, sex and strain in a series of previous experiments using dye injections and subsequent brain sectioning [9, 16]. During a postoperative recovery period of 7–10 days, the rats were weighed and gently handled daily. After this, the cannula placements were assessed by a functional test. The animals were injected with 210 μg 5-thio-d-Glucose (5TG) into the fourth ventricle, and an increase in blood glucose which exceeded 100% from baseline was taken as a correct placement [17]. One animal did not respond to 5TG, and was therefore not included the study. The animals were handled daily for 1 week following cannula placement testing, but did not undergo any other procedures.

### Drugs

Synthetic CART(55–102) peptide (rat; American Peptide, Sunnyvale, CA) and α-Helical CRF (9–41) (Sigma-Aldrich) were dissolved in saline and distilled water, respectively. CRF (Sigma-Aldrich) was dissolved in saline. The respective solvents were also used as the corresponding vehicles. The dissolved drugs were aliquoted and frozen (− 20 °C). Fresh aliquots were thawed on each experimental day and the excess was discarded.

### Fourth Intracerebroventricular injections

The animals were gently restrained by hand, and a 32-G injection needle was inserted in the guide and into the fourth ventricle. The injection needle was attached to a Gilmont microinjector via a 20 PE tube, and 1.5 μl of vehicle or drug was injected into the fourth ventricle over 1 min, such that a total volume of 3 μl was administered. The needle was left in place for another 45 s to avoid the risk of back flush, after which it was removed and replaced with an obturator. Finally, the animal was returned to its home cage.

## Experimental design

Food intake experiments were performed every third day to allow for any carry-over effects of drugs to be washed out. The study had a 3Rs reduction design such that animals (n = 9 in each experiment) served as their own controls, and each animal received each combination of drugs once and in random order, so that any possible carry over effects could be avoided. No exclusions were made. Food intake monitoring was done in the home cage. The animals had free access to food until 1 h before lights off, when the food hoppers were removed. Drug injections were administered and the pre-weighed food hoppers were returned to the respective home cage at lights off. The food was thus weighed before lights off/food presentation and after 2 h, 4 h and 22 h of food access. The dry weight food intake was calculated with correction for food spillage collected on metal trays under each cage, at each time point.

In *experiment 1*, the effect of pre-treatment with the non-selective CRF antagonist αCRF on CARTp-induced feeding suppression was investigated. The un-selective CRF receptor antagonist alpha-helical-CRF9-41 was used for two reasons, first, since it has previously been shown effective to counteract gastrointestinal effects of CRF as well of CARTp after fourth i.c.v. administration at the dose used here, without displaying any effects by itself. Second, because a subtype-selective antagonist only has affinity for one or some receptor subtypes, and a competitive antagonist acts by displacing the agonist from the receptor. An agonist displaced from one receptor subtype could then theoretically increase its binding to a different subtype of the receptor, which the subtype-selective antagonist does not have affinity for. This could result in a confounding agonistic effect which is less likely to occur if an un-selective antagonist is used. Fourth i.c.v. injections of vehicle or αCRF, followed by vehicle or CARTp, were given 30 and 10 min prior to lights off. Vehicle, CARTp (0.5 μg or 1 μg) and/or αCRF (10 nmol) was injected. The following drug combinations were given: vehicle + vehicle, vehicle + 0.5 μg CARTp, vehicle + 1 μg CARTp, αCRF + vehicle, αCRF + 0.5 μg CARTp, and αCRF + 1 μg CARTp, respectively.

*Experiment 2* was a control experiment aimed at verifying the antagonistic effect of fourth ventricular αCRF versus its agonist, corticotropin-releasing factor, on food intake inhibition. The drug and dose combinations administered in experiment 2 were: vehicle + vehicle, vehicle + 1 nmol CRF, 10 nmol αCRF + vehicle, and 10 nmol αCRF +1 nmol CRF, respectively. Drugs were administered into the fourth ventricle at similar time points and in similar volumes as described for experiment 1. The respective doses were chosen based on previous publications showing efficacy in inducing (CRF) and reversing (CRF antagonist versus CRF) effects of food intake, respectively.

### Data evaluation

For both experiments, the cumulative food intake results were evaluated using repeated measures ANOVA at each time point (2 h, 4 h, and 22 h), followed by Fischer’s PLSD test for post hoc comparisons. Statistica v. 7.1 software (Statsoft Scandinavia AB) software was used for statistical evaluation.

## Results

In experiment 1, repeated measures ANOVA’s showed significant overall effects of treatment at each of the three time points; (at 2 h: F_5,8_ = 10.734, *p* < 0.0001; at 4 h: F_5,8_ = 8.330, *p* < 0.0001 and at 22 h: F_5,8_ = 5.556, *p* < 0.001). Post hoc analyses revealed a significant effect of 1 μg CARTp to reduce food intake at all the three time points (vehicle/CARTp condition vs. vehicle/vehicle condition: *p*’s < 0.001, respectively). After injection of 0.5 μg CARTp (vehicle/0.5 μg CARTp condition), food intake for each of the time points appeared numerically somewhat lower versus control (vehicle/vehicle condition), but there were no significant effects, supporting the suggestion of 0.5 μg CARTp as a subthreshold fourth i.c.v. dose for food intake suppression.

In addition, αCRF had no effect by itself on food intake (*p* > 0.05, ns: αCRF/vehicle vs. vehicle/vehicle conditions). In combination with αCRF pre-treatment, the subthreshold CARTp dose (0.5 μg) significantly suppressed food intake at 2 h (*p* < 0.05: vehicle/0.5 μg CARTp vs. αCRF/vehicle condition as well as vs. vehicle/0.5 μg CARTp condition). At 4 h and 22 h, however, the feeding inhibition induced by the combination of a low, subthreshold dose of CARTp (0.5 μg) and αCRF pre-treatment was no longer significant (*p*’s > 0.05, ns: αCRF/0.5 μg CARTp vs. αCRF/vehicle.

**Fig. 1:**
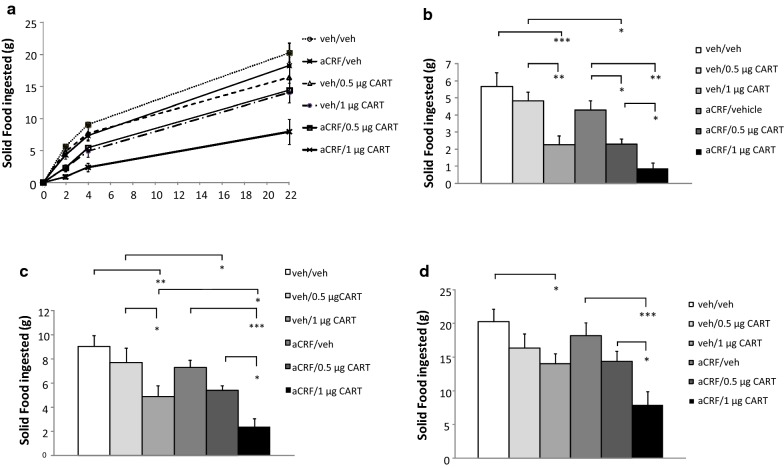
Pretreatment with the CRF antagonist effectively increased efficacy of CARTp-induced feeding suppression. **a** Effects over time of a subthreshold dose, and an effective dose of CARTp, αCRF, or CARTp after αCRF pretreatment, on ad libitum solid food intake over 22 h. Results at each separate time point are shown in panels **b**–**d**; **b** food intake effects of 0.5 or 1 µg CARTp, 10 nmol αCRF, αCRF and respective CARTp-dose, or vehicle after 2 h, **c** 4 h and **d** 22 h (*NS* not significant; *p < 0.05; **p < 0.01; ***p < 0.001)

aCRF pre-treatment significantly amplified the feeding suppression produced by 1 μg CARTp but in contrast to the subthreshold CARTp dose, this effect was sustained throughout the 22 h testing period (αCRF/1 μg CARTp vs. αCRF/vehicle at 2 h: *p* < 0.01 and at 4 and 22 h: *p*’s < 0.001; and αCRF/1 μg CARTp vs. vehicle/1 μg CARTp at all three time points: *p*’s < 0.05, respectively). Moreover, the suppression was dose dependent (αCRF/1 μg CARTp vs. αCRF/0.5 μg CARTp, *p* < 0.05 for all the three time points) such that αCRF pre-treatment caused a right shift of the CARTp responsivity dose curve (Fig. 1a). The results from exp. 1 are displayed in Fig. 1.

**Fig. 2:**
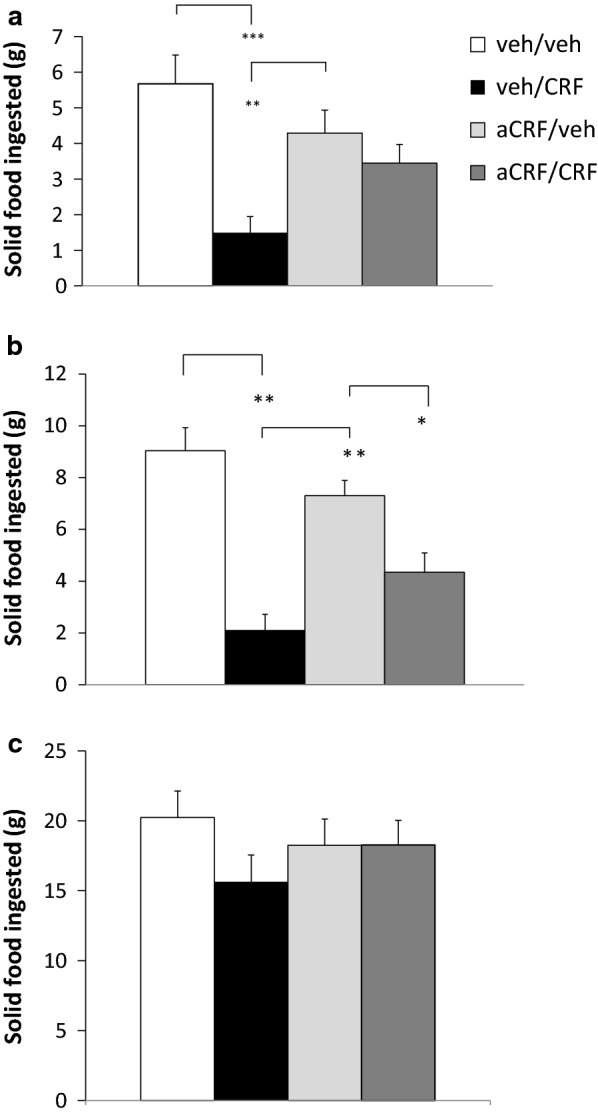
Antagonistic effect of αCRF (10 nmol) versus a high dose (1 nmol) of CRF agonist. Drugs and/or vehicle were administered fourth i.c.v. and solid food intake was measured at **a** 2 h, **b** 4 h and **c** 22 h, respectively. CRF suppressed food intake at 2 h and 4 h, respectively. This was blocked by αCRF, which did not produce any significant effect by itself at any time point, thus confirming the efficacy of the CRF antagonist in the current paradigm (*NS* not significant; *p < 0.05; ***p < 0.001)

In experiment 2, there was a significant main effect of CRF treatment after 2 and 4 h, but not after 22 h (F_3,8_ = 8,125, *p* < 0.001; F_3,8_ = 22,266, *p* < 0.001; and F_3,8_ = 1.050, *p* > 0.05, ns, respectively). Fischer’s PLSD test showed a significant effect of 4th i.c.v. CRF (*p* < 0.001) (vehicle/1 nmol CRF vs. vehicle/vehicle) on solid food intake at 2 h and at 4 h. This was blocked by αCRF pre-treatment (αCRF/CRF vs. vehicle/CRF, *p* < 0.05) at 2 h and 4 h. There was no significant effect by αCRF by itself on food intake (αCRF/vehicle vs. vehicle/vehicle condition) at any time point. The results from exp. 2 are shown in Fig. 2.

## Discussion

In the present study we sought to evaluate the hypothesis that the anorexic response to brainstem CARTp administration requires activation of a CRF pathway. The plausibility of this hypothesis is suggested by the fact that inhibitory effects of both feeding and gastric emptying are obtained with the same fourth i.c.v. placements [3, 7, 14, 16] and that the emptying effect as shown previously was completely reversed by pre-treatment with the un-selective CRF antagonist αCRF [4, 7, 16]. The question of common versus separable substrates underlying the feeding and/or gastro-motor effects, respectively, was of interest partly because local nano-injections of CARTp into the dorsal vagal complex showed positive result for the emptying effect [5] but not for feeding [6]. Further, the possibility that CRF-mediation may apply to the emptying effect but not to the feeding effect arose from the failure of αCRF to antagonize CARTp-induced intake of sucrose in the rat [7]. We replicated previous effects of fourth i.c.v. CARTp on solid food intake inhibition, here in the rat ingesting solid food in the dark phase. We further showed in the similar feeding paradigm that the CRF antagonist dose chosen for this study was sufficient to fully block the effects of CRF on feeding after fourth i.c.v. application, which at the agonist dose chosen yielded an anorexic inhibitory response of comparable magnitude to that seen with CARTp administration (exp. 2). However, rather than block the feeding inhibitory effects of fourth i.c.v. CARTp, the non-selective CRF antagonist enhanced it. The results of the critical experiments here thus lead us to firmly reject the CRF-receptor activation hypothesis for CARTp on food intake.

**Fig. 3:**
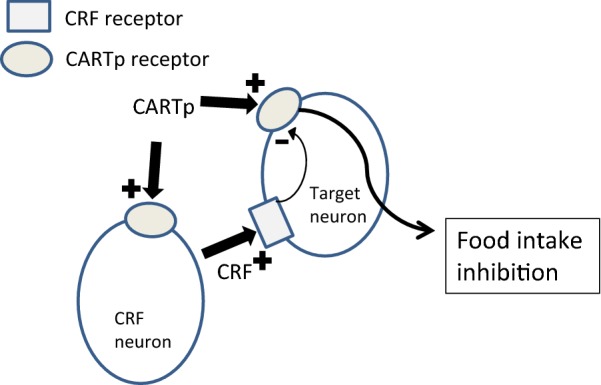
A hypothetical auto-modulatory caudal brainstem mechanism for CARTp–CRF receptor interactions to affect feeding. We raise the hypothesis that CARTp may inhibit food intake via a putative receptor on a target neuron at some level in the dorsal hindbrain. CARTp could thus act via a CRF intermediate, which, via tonic lateral inhibition or perhaps via an inhibitory interneuron mechanism, attenuates the CARTp hypophagia. In the absence of stress activation, addition of a CRF antagonist would not by itself impact feeding but simply block CRF receptors, resulting in the loss of its tonic inhibition of the CARTp and subsequently in the unmasking of a full CARTp hypophagic behavioral response

There was indeed a significant interaction between CARTp and CRF with respect to feeding, but the form of the interaction was unexpected, and to our knowledge, unprecedented. It would have been unremarkable if αCRF had simply failed to reverse the feeding effect of CARTp, however the CRF antagonist in fact, strongly amplified, the CARTp effect. The strongest inhibition observed in the study was with αCRF injected before an effective dose of CARTp. Further evidence of an additive or synergistic interaction can be seen from the significant reduction of intake from baseline levels when αCRF was given prior to a CARTp dose that was subthreshold for the feeding response. There are some evident conclusions that can be drawn from the present results. First it can be clearly stated that the CARTp triggers for feeding and gastro-motor responses may be anatomically distinct, or at least partially non-overlapping. Second, we confirm that the mediating substrates for the respective responses are clearly distinguishable at least as shown with this functional probe of CRF involvement [7]. The antagonist alone did not significantly affect feeding, suggesting that basal CRF agonist activity is not involved in the feeding response under normal physiological conditions. The results imply that CARTp in fact drives a CRF pathway, and that this pathway exerts inhibitory action on downstream target cells that also respond to CARTp resulting in an inhibitory net effect on feeding. A schematic illustration of this proposed mechanism is shown in Fig. 3.

Both CARTp and CRF in the hypothalamus can affect food intake. In addition, they are associated with the stress response, via activation of the hypothalamic–pituitary–adrenal axis. In this study, the focus was not on hypothalamic effects however, but on mechanisms elicited at a dorsal hindbrain level. Whereas it could be tempting to view effects by endogenous CRF-receptor agonists in the dorsal hindbrain as well as simply stress-associated, our present data suggest that these compounds may in fact interact and contribute to modulation of feeding behaviour also under normal unstressful conditions. Our paradigm involved animals that were well habituated to handling and the testing procedure and care was taken not to interfere with normal routines unless necessary in order to avoid any confounding stressful events. Animals were adjusted to being removed from the home cage and receiving drug injections only for a brief moment, and then returned to the home cage where the recording of its nocturnal ad libitum food intake was done with minimal disturbances. The lack of significant effects on food intake [14, 16] by the CRF antagonist, supports the notion of a testing situation absent of stress activation. The observed effect of CARTp may thus be interpreted as part of a normal behavioral response to a CARTp—endogenous CRF receptor agonist interaction, and not a consequence of stress activation.

In order to draw any more specific anatomical conclusions regarding the regulatory circuitry for the CARTp/CRF effects on feeding, the discussion becomes speculative due to the fact that the receptor locations for CARTp are yet to be defined. As outlined in the Introduction, CART is present in several dorsal hindbrain nuclei that are involved in food intake control, which also express receptors for CRF, endogenous CRF receptor agonists or are innervated by CRF-containing fibers. After local injection in the NTS, CARTp did not affect food intake in rats [6]. It is clear however, that CARTp does have strong food intake inhibitory effects at a dorsal hindbrain level [3, 5, 13] and that CARTp acts in the dorsal vagal complex to inhibit gastric emptying via a vagal efferent pathway [5]. The dorsal hindbrain effects of CARTp on gastric emptying [7], gastric acid secretion [4, 5] and of c-fos expression in the dorsal vagal complex [9], each appear to be mediated via an endogenous CRF receptor agonist intermediate. It is plausible that relevant CRF receptors contribute to the different responses within the field of infusion, i.e., that each act within the dorsal hindbrain, and in proximity of the fourth ventricle, at close but not similar targets. By comparing the effects of CARTp with and without pre-treatment with αCRF on gastric emptying, it is clear that the threshold dose for gastro-motor inhibition is lower (0.5 μg) as compared to the threshold dose for food intake inhibition (1 μg) [2, 7, 13]. Together with the present finding that a CRF receptor mediated mechanism action may cause attenuation of CARTp-induced food intake inhibition, this suggests that the hindbrain controls for gastric emptying are not only separate, but express differences in sensitivity and in the principle mechanism of control.

Future investigations may address whether these findings translate to other species or systems, including humans.

## Perspectives

A main finding of the present study was that pre-treatment with αCRF resulted in an apparent increased efficacy of CARTp. Together with previous reports on CARTp- and CRF interactions in the dorsal hindbrain, this opens the possibility that CARTp effects on feeding and gastrointestinal functions are modulated by an endogenous CRF agonist downstream not only through separable controlling pathways, but via separate regulatory mechanisms as well. CARTp inhibits food intake via a caudal brainstem mechanism for which the precise neuronal sites of action are not yet known. CRF receptor antagonism attenuated the CARTp-induced inhibition of food intake as shown here. As schematically illustrated in Fig. 3, we present the hypothesis that the CARTp effect discovered, could be auto-modulated by an endogenous CRF agonist-induced lateral inhibition mechanism. Blocking of the CRF receptor as in the present study would thus release a “CRF brake” on CARTp, resulting in an increased net efficacy of CARTp under this paradigm. The behavioral end result is an increased CARTp-induced inhibition of food intake. This notion would explain why αCRF does not produce any apparent effects per se under un-stressful conditions, since the effect only can be detected when the “CARTp-brake” is off, as is the case when CRF receptors are blocked in the presence of CARTp. The ability for CARTp to induce CRF not only in the hypothalamus [8], but at a dorsal hindbrain level as well is implied by the observation that delayed gastric emptying after fourth i.c.v. CARTp was inhibited by αCRF [7], as was expression of c-fos [9]. There are mechanisms by which agonist-receptor interactions may modify the sensitivity of the specific receptor. In 1998, a new group of receptor activity modifying proteins (RAMPs) that are present in the cell membrane and act to modify receptor activity and function were described [18]. RAMPs interact with several G-protein-coupled membrane peptide receptors including the CRF1 receptor and the VPAC1 and − 2 receptors and modulate their function, sensitivity and expression. Activation of CRF1 receptors in vitro was shown to enhance expression of RAMP2, and the RAMP2:CRF1 receptor complex increased CRF1 receptor sensitivity to CRF and urocortin by elevation of intracellular calcium [19]. Whether RAMPs could contribute to such “lateral inhibition” as proposed here by changing the responsiveness to CARTp after CRF-receptor antagonism in vivo remains to be investigated.

## Conclusions

Pre-treatment with a CRF antagonist, while having no significant effect by itself, increases the efficacy of CARTp to inhibit solid food intake at a dorsal hindbrain level. This indicates involvement of a caudal brainstem mechanism by which endogenous CRF activity can strongly attenuate CARTp food intake inhibition under normal, unstressful conditions in the rat.

## Abbreviations

αCRF: α-helical CRF9-41
CRF: corticotropin-releasing factor
CARTp: cocaine-and amphetamine-regulated transcript peptide
Fourth i.c.v.: into the fourth cerebral ventricle
i.c.v.: intracerebroventricular
i.c.: intracisternal
i.m.: intramuscular
i.p.: intraperitoneal
5TG: 5-thio-d-glucose
RAMPs: receptor activity modifying proteins
